# Alcohol use among populations with autism spectrum disorder: narrative systematic review

**DOI:** 10.1192/bjo.2024.824

**Published:** 2025-01-13

**Authors:** William Barber, Betul Aslan, Tim Meynen, John Marsden, Samuel R. Chamberlain, Vigneshwar Paleri, Julia M.A. Sinclair

**Affiliations:** Addictions Department, School of Academic Psychiatry, Institute of Psychiatry, Psychology and Neuroscience, King's College London, London, UK; Department of Psychiatry, Faculty of Medicine, University of Southampton, Southampton, UK; Department of Psychiatry, Faculty of Medicine, University of Southampton, Southampton, UK; and Southern Health NHS Foundation Trust, Southampton, UK

**Keywords:** Autistic spectrum disorders, alcohol disorders, comorbidity, mental health services, neurodevelopmental disorders

## Abstract

**Background:**

Alcohol use in autism spectrum disorder (ASD) is under-researched. Previous reviews have explored substance use as a whole, but this neglects individual characteristics unique to different substances. Alcohol use in non-clinical samples is associated with diverse responses. To advance practice and policy, an improved understanding of alcohol use among people with ASD is crucial to meet individual needs.

**Aims:**

This was a narrative systematic review of the current literature on the association between alcohol use and ASD, focusing on aetiology (biological, psychological, social and environmental risk factors) and implications (consequences and protective factors) of alcohol use in autistic populations who utilise clinical services. We sought to identify priority research questions and offer policy and practice recommendations.

**Method:**

PROSPERO Registration: CRD42023430291. The search was conducted across five databases: CINAHL, EMBASE, MEDLINE, PsychINFO and Global Health. Included studies explored alcohol use and ASD within clinical samples.

**Results:**

A total of 22 studies was included in the final review. The pooled prevalence of alcohol use disorder in ASD was 1.6% and 16.1% in large population registers and clinical settings, respectively. Four components were identified as possible aetiological risk factors: age, co-occurring conditions, gender and genetics. We identified ten implications for co-occurring alcohol use disorder in ASD, summarised as a concept map.

**Conclusion:**

Emerging trends in the literature suggest direction and principles for research and practice. Future studies should use a standardised methodological approach, including psychometrically validated instruments and representative samples, to inform policy and improve the experience for autistic populations with co-occurring alcohol use.

Autism spectrum disorder (ASD) is a neurodevelopmental condition characterised by social communication/interaction challenges, restrictive and repetitive behaviour, inflexible behaviour patterns and atypical or excessive interests or hobbies.^1^ ASD is usually taken to capture a broad range of disorders, which has been previously referred to as Asperger's syndrome, pervasive developmental disorder and autism spectrum disorder. There is no universally accepted consensus on the terminology used to describe ASD. Here, we use identity-first language (‘autistic person/people’^2^) as this has been recommended by the stakeholders of the Substance use, Alcohol, and Behavioural Addictions in Autism partnership (SABA-A^3^).

A recent UK study has suggested progressive increases in ASD diagnoses over time, with the greatest increase in adults and women.^4^ The apparent growth in the prevalence of ASD has been attributed to changes in diagnostic criteria,^5^ reporting practices,^6^ and increased awareness.^7^ Despite suggested increases in ASD diagnoses, waiting time for assessment continues to rise across Europe.^8^ Consequently, many autistic people continue to access healthcare services unrecognised and as incident rates increase,^9,10^ so does the need to explore further co-occurring conditions to improve treatment outcomes.

Within ASD populations, research indicates alcohol is the most reported substance used.^11–13^ Previous reviews and meta-analyses of substance use disorder (SUD) in autistic populations reported a wide prevalence range of alcohol-use disorder (AUD; 0–16%), attributed to heterogeneity across study samples and the diagnostic procedures used.^11,12,14,15^ Several studies have found when compared with neurotypical peers, autistic adults report lower alcohol use and higher rates of abstinence.^16–18^ Further findings from two studies showed that the rate of alcohol use in autistic people increases with age.^18,19^

AUD is a spectrum disorder characterised by maladaptive patterns of alcohol use and related impairments of personal and social functioning that are clinically assessed as mild, moderate or severe.^20^ As criterion symptoms accumulate, the risk of harm and adverse psychosocial consequences increases.^21^ Of all SUDs, AUD is the most prevalent within global populations, requiring targeted interventions and policies to reduce alcohol-related harm.^22,23^ Of note, the language used to describe AUD may be stigmatising, leading to significant barriers to receiving or seeking support.^24^ Here, we extracted the same phraseology used in studies, but we omitted stigmatising labels such as addict, misuse and abuse within the main body.

## Biopsychosocial factors of AUD and ASD

A greater understanding of both risk and protective factors in ASD could improve translational opportunities for research and clinical practice.^25^ Despite the estimates mentioned previously, the prevalence and nature of AUD among autistic people are underexplored.^3,11^ From a biopsychosocial lens, identifying shared factors between conditions can offer avenues to promoting resilience and intervention of risk factors.

Typically, the developmental onset and course of both ASD and AUD are diverse. Early conceptualisations position ASD as emerging in childhood,^26^ while AUD routinely develops across the lifespan from adolescence and early adulthood.^27,28^ Both conditions possess an element of genetic heritability.^29,30^ Two reviews found an overlap of neurological circuitry between ASD and SUD,^31^ as well as several independent studies which observed overlapping genes in the susceptibility to ASD or AUD.^32^ These findings suggest the possibility of shared genetic pathways between both conditions. However, the identification of specific risk variants for each remains inconclusive and emphasises consideration for epigenetic interactions.^33–35^

Several environmental factors are known to contribute to the development of AUD; including parental alcohol use and supply,^36^ low prosocial behaviours,^37^ social norms,^38^ peer substance use,^39^ and adverse childhood experiences.^40^ Research has found some autistic people may have an equal or greater likelihood of experiencing traumatic events compared with neurotypical peers.^41–43^ This connection could be attributed to interpersonal victimisation and bullying, emotional dysregulation in processing traumatic stress, lack of support and social isolation common in ASD.^41–43^ Exposure to traumatic events, as a common factor for both ASD and AUD, may increase the overall risk of harmful alcohol use, whereas social capital, referring to the level of community attachment, closeness and supportiveness experienced by an individual, has the potential to reduce the risk of alcohol use.^44^ However, as mentioned previously, social isolation and lack of support are common in autistic people, presenting social capital as a possible area of vulnerability.

Characteristic features of ASD may be protective, influencing how an autistic person interacts with their environment. For example, developmental and communication challenges, as well as unsettled peer relationships in ASD adolescents, were negatively associated with alcohol and substance use.^45^ In addition, certain factors such as parental involvement, household rules and monitoring can limit the availability and opportunity for alcohol use among autistic individuals, thus reducing the risk of developing AUD.^39,46^

Bowri et al^17^ examined factors associated with alcohol use within a high-functioning community sample of autistic adults. Dividing the sample into three groups by alcohol use, non-drinkers and hazardous drinking patterns were predictors for higher scores of autistic traits, depression, social anxiety and generalised anxiety, in comparison with non-hazardous drinkers. Non-hazardous drinkers reported the highest scores of well-being among the three groups, whereas hazardous use was associated with a higher frequency of co-occurring psychiatric conditions. However, these findings did not distinguish whether alcohol use acts as a protective factor against co-occurring psychiatric conditions or if reduced co-occurring conditions led to less harmful alcohol use.

Antecedents to alcohol use have focused on positive and negative motivations for use.^47–49^ Social facilitation, mood enhancement, symptom management, coping mechanisms for difficulties such as social anxiety, sensation seeking and ‘self-medication’ of sensory processing difficulties are themes positively associated with alcohol use.^16,47–51^ In contrast, factors such as fear of addiction, disinhibition, olfactory sensitivity and decreased access to alcohol limit the risk and decrease motivation for alcohol use in ASD populations.^17,47,52^ Given the diverse levels of functioning and severity, inherent within the spectrum of both ASD and AUD, current research lacks a validated measure that simultaneously captures elements of both conditions. This absence of standardisation across studies impairs the ability to establish consistent conclusions from findings.^3^ Overall, there is a greater need to increase screening and prevention, and to reduce barriers to support for autistic people with AUD.^47,53–55^

## Current review

The Substance use, Alcohol, and Behavioural Addictions in Autism partnership (SABA-A), funded by the Society for the Study of Addiction, brought together a range of experts to identify key policy, research and clinical practice questions for ASD and addiction. In 2023, the project published the top ten priorities, outlining the most urgent issues impacting the lives of autistic individuals with substance use, problematic alcohol use or behavioural addictions.^3^ The highest-ranked priority was the identification and prevention of specific triggers, risk factors and facilitators of substance, alcohol or behavioural addictions in autistic people. Additional priorities included enhancing awareness, reducing stigma, adaptations to current approaches, how other conditions or traits impact the development and maintenance of addiction and differences in vulnerability between autistic and non-autistic populations. SABA-A has published one review on ASD and gambling and the current review explored ASD and alcohol use.^56^

Existing reviews have generally focused on SUD, rather than exclusively on AUD. Different substances can serve different purposes for autistic adults, and given that alcohol use is common within ASD populations, it is of interest that there is limited information on how ASD and AUD present in clinical services.

Accordingly, our aim was to:

1. systematically identify and collate findings from studies which have examined the association between AUD and ASD;
2. explore the current knowledge of clinical samples on aetiology, including biological, psychological, social and environmental risk factors associated with alcohol use among autistic people; and implications, including the protective factors and consequences of co-occurring alcohol use in autistic people;
3. identify priority research questions and offer recommendations for policy and clinical practice.

## Method

### Protocol registration

This was a pre-registered systematic review. The protocol was prospectively registered with the International Prospective Register of Systematic Reviews (PROSPERO) on 31 May 2023 (identifier CRD42023430291).

### Design and search strategy

We conducted a search of the extant literature using five databases: CINAHL, EMBASE, MEDLINE, PsychInfo and Global Health. The search string consisted of: ((ASD) OR (Autis*) OR (Asperger*) OR (ASC) OR (PDD) OR (Pervasive Developmental Disorder) OR (Neurodevelopmental) OR (Kanner) OR (Developmental Disabilities / OR Autism Spectrum Disorders / OR Child Developmental Disorders [MeSH Major Topic])) AND (Alcohol*) OR (Alcohol addiction) OR (Alcohol misuse) OR (Alcohol dependen*) OR (Drinking) OR (Alcoholism / OR Alcohol abuse / OR Alcohol Use Disorder / OR Alcohol related disorders [MeSH Major Topic])). This string was adapted from an initial scope conducted by one of the authors (B.A.) and finalised with current authors.

The search was completed as planned with the exception at full-text screening to narrow the search to clinical samples only. Clinical samples were defined as participants recruited from healthcare settings or patient register databases. Given the heterogeneity of existing literature, this approach was taken to provide future clinical services and research recommendations as a meta-analysis would not be feasible at this stage. The search was repeated in August 2023 to identify any additional papers since the initial search in May 2023. This review adhered to narrative synthesis and PRISMA guidelines (see Supplementary material for checklist).^57,58^

### Inclusion and exclusion criteria

Primary inclusion required studies to explore broadly both ASD and alcohol use in a clinical setting or population. Studies that measured autistic traits were also included. We applied no limits on the date of publication or age of participants, accounting for potential longitudinal studies. Both qualitative and quantitative articles were included to capture reported life experiences alongside statistical inferences and associations between ASD and alcohol use. Included studies were required to be peer reviewed and published in English. We excluded studies of other reviews, meta-analyses, studies with an ASD sample of less than five, genome-wide association studies, book chapters and grey literature.

### Data extraction and synthesis

A narrative synthesis was the preferred method of analysis as the literature is limited. Articles were initially screened by title and abstract, using the criteria specified, before retrieval for full-text review. Where studies did not solely focus on ASD and alcohol use, only related data were extracted. The first data extraction took place on 16 June 2023 and final data extraction on 18 August 2023, following the repeated search. Two raters (W.B., V.P.) were used at both steps of the screening process and quality appraisal. Any discrepancies were discussed among the authors until a consensus was reached. The overall approach was guided by the Popay et al^58^ framework for narrative synthesis. Tabulation was used to infer similarities across studies, and themes were grouped across aetiological factors and potential implications of ASD and alcohol use. The summary of findings is presented as a concept map, to capture key themes and inform the third aim, identifying priority areas and recommendations.^58^

### Quality assessment

The Mixed Methods Appraisal Tool (MMAT^59^) was used to assess the methodological quality of selected studies. Each study was evaluated by design and rated across five criteria, individual to each study design (yes, 1; no, 0), with an overall quality score calculated as a percentage of total criteria met (presented in Table 1^60^). We did not set a minimum threshold for quality criteria and, therefore, did not exclude any studies. Full quality description can be found (Supplementary Table 2 available at https://doi.org/10.1192/bjo.2024.824).

**Table 1:** Summary of studies included in the review

Study	Quality assessment^a^	Population/setting	Design and sample size	AUD measure	Age^b^	Gender	Race/ethnicity	Socioeconomic^c^	Key findings (Sig. is statistical significance)
**AUD service**									
Hildebrand Karlén et al (2021)^61^	40%	Alcohol treatment settings (inpatient and outpatient)	Longitudinal, quantitative non-randomised (*n =* 153; *n =* 91 completed 2.5 year follow-up)	Previous dx of AUD based on DSM-IV criteria. Additional structured interview of ASI	M = 50.2 (8.56) 29–71	80.8% male	NR	11.01 years in school; 87.05% in current employment; 57.3% in current relationship	Sig. but weak correlations between autistic personality traits (APTs) and baseline consumption (positive) and APTs and age at entering treatment (negative). Post-treatment, the number of APTs was not correlated with (1) how much participants lowered or maintained consumption, (2) whether they normalised drinking or (3) were neither more nor less prevalent in abstainers. Problematic drinking patterns 2.5 years after treatment were sig. related to idiosyncratic features of ASD.
Kronenberg et al (2014)^62^	100%	Outpatient dual diagnosis service. Treatment-seeking individuals with ASD or ADHD and comorbid SUD	Qualitative interviews (*N* = 23; ASD + SUD *n =* 12)	Previous dx of SUD based on DSM-IV criteria.	ASD+SUD sample: M = 37 (NR)	ASD+SUD sample: 100% male	NR	ASD+SUD sample: Employment: 50% employed; Living: 42% living alone, 58% living with others	Three main themes: (1) jumbled thoughts and emotions, (2) ambiguity of substance use and (3) structure.
Narita et al (2016)^63^	80%	Hospital patients with AUD	Case-control, quantitative non-randomised cohort (*N* = 139, AUD *n =* 64, Unrelated controls *n =* 75)	Previous dx based on DSM-IV criteria	AUD: M = 57.3 (10.18) NR	AUD: 78.1% male	NR	All patients lived in Yamagata prefecture, Japan	No sig. difference between the AUD group and control group for autism susceptibility candidate 2 gene (AUTS2) polymorphisms. Distribution of A-A haplotype combinations were sig. different and higher in frequency within the AUD group compared to controls.
Walhout et al (2022)^64^	60%	Addiction treatment centre.	Naturalistic, quantitative non-randomised cohort (T0 *N =* 57, T1 *n =* 30, T2 *n =* 27)	Previous SUD dx based on DSM-5 criteria. Additional measure of MATE 2.1	M = 36.8 (11.65) 19–64	86% male	Country of birth: 93% Netherlands, other European 3.5%, non-European 3.5%	Education: 33.3% ‘Low’, 43.9% ‘Medium’, 21.1% ‘High’; Employment: 75.4% unemployed/ social benefit, 24.6% Job/ student loan; Marital status: 71.9% single, 17.5% married, 10.5% divorced/ widow/ widower	Sig. decrease in alcohol consumption from baseline to time 1 and time 2 following adapted group cognitive behavioural therapy (CBT) for SUD. Cannabis use remained unchanged at both time points. Sig. decrease in cravings, passive coping style and symptoms of depression, anxiety and stress at time 1 and 2. Sig. increase in seeking social support as a coping mechanism, overall feelings of control and self-empowerment at time 1 and 2. At time 2, there was a sig. increase in using reassuring thoughts, learning new potentials and spirituality.
Yoshimura et al (2022)^65^	100%	Addiction treatment centre	Naturalistic longitudinal, quantitative non-randomised cohort (*N =* 637)	Clin. ax. using ICD-10 criteria	M = 53.9 (12.9) 20–85	85.2% male	NR	NR	The presence of ASD traits (*n =* 29) did not affect abstinence rates during follow-up compared to those without traits (*n =* 461). When divided into three groups based on autism-spectrum quotient (AQ, Low, moderate, High), no differences were observed in abstinence rates.
**ASD service**									
Miles et al (2003)^66^	20%	Unrelated patients with infantile ASD were recruited from university hospital and clinics	Family history interview, quantitative non-randomised (*n* = 167; split high alcoholism *n* = 65, low alcoholism *n* = 102)	Semi-Structured Assessment for Genetics of Alcoholism (SSAGA)	High alcoholism family: 1.1–39.8 Low alcoholism: 1.0–41.2	81.4%	NR	Socioeconomic status (Hollingshead scale): High alcoholism: 36% group I & II, 28% group III, 36% group IV & V; low alcoholism: 50.6% group I & II, 25.8% group III, 23.6% group IV & V	Prevalence of alcoholism in first- and second-degree relatives was 13.7% of ASD probands. Males (20.3%) were sig. more likely to have a history of alcoholism than females (6.6%). 39% (*n* = 65/167) met the criteria for alcoholism pattern consistent with the study criteria for genetic trait. In high alcoholism families, females were 18 times more affected (38.2% v 8.7%) and males four times (15.9% v 0.9%) than in low alcoholism families. Ratio of female to male alcoholism was sig. higher in high-alcoholism families compared to low alcoholism.
Roy et al (2015)^67^	60%	Outpatient clinic seeking AS diagnosis	Cross-sectional, quantitative non-randomised cohort (*N* = 50)	SCID-I (DSM-IV)	M = 36.5 (NR) 20–62	68% male	NR	Income: 42% own income, 50% financial support, 8% disability pension; Education: 4% ‘none’, 4% ‘special school’, 12% ‘low’, 22% ‘intermediate’, 58% ‘high’; Employment: 46% currently employed, 52% not employed; Living: 48% live alone, 22% with a partner and/or child, 28% live with parents, 2% live in psychiatric nursing	Alcohol abuse or dependence was present in 18% of the population (*n* = 9). Harmful AUD/dependence prevalence was unequal across genders (males 20%: females 12%). Prevalence of alcohol dependence (8%) was higher than a German population sample (6.3%). Age differences were also found, with alcohol dependence only present in elderly individuals (≥40 years).
Yule et al (2023)^68^	100%	Medical chart review from specialised ambulatory programme for ASD	Retrospective case-control, quantitative non-randomised (*N* = 679; ASD *n* = 230, ADHD *n* = 219, controls *n* = 230)	Clin. ax. using DSM-III-R and DSM-IV for SUD	ASD sample: M = 20.0 (10.3) 12–59	79% male	93% Caucasian, other ethnicities NR	Socioeconomic status was measured using the Hollingshead scale. ASD sample: M = 2.0 (1.0)	ASD sample had sig. higher rates of bipolar disorder, major depressive disorder, multiple anxiety disorders, oppositional defiant/antisocial personality disorder and conduct disorder compared to controls. Non sig. prevalence of AUD in comparison to controls but sig. lower than participants with ADHD. Sig. lower risk of developing AUD in ASD than ADHD and non-sig. but a downward trend with controls. ASD participants were sig. older when they developed AUD compared to ADHD or controls.
**ASD and AUD service**									
Clarke et al (2016)^69^	80%	Asperger syndrome service; Drug and alcohol service	Qualitative interviews (*n* = 8)	SUD dx based on DSM-IV criteria; combined with DAST and AUDIT	M = 35.4 (14.65) 21–55	87.5% male	NR	NR	Six themes for ‘factors contributing to substance use’: social facilitation, self-medication, recreational use of substances, substance use of peers, defining problematic substance use and a discrepancy between need and support. Subthemes: substance used to increase social confidence; substances used to facilitate communication.
**Forensic service**									
Anckarsäter et al (2008)^70^	40%	Group 1 – Special psychiatric hospital inpatients; Group 2 – forensic psychiatry; Group 3 – special institution for adolescents	Case-series, Quantitative non-randomised (Total *n* = 42; Group 1 *n* = 4, Group 2 *n* = 18, Group 3 *n* = 20)	Medical note review (group 1&3) and structured interviews (group 2)	Group 1: Mdn = 27 (19–46) Group 2: Mdn = 25.5 (18–47) Group 3: Mdn = 15 (11–18)	Group 1: 50% male Group 2: 83.33% male Group 3: 70% male	NR	NR	Prevalence of AUD in individual ASD diagnoses: autism *n* = 2/5, atypical autism *n* = 3/10. Prevalence within group 2 only 27.78%.
Chaplin et al (2021)^71^	60%	Male prisoners	Cross-sectional, Quantitative non-randomised (*n* = 240; *n* = 46 screening positive for ASD traits, of these *n* = 12 meeting Autism Diagnostic Observation Schedule (ADOS) criteria)	MINI for dx based on ICD-10 criteria	Positive ASD traits (*n* = 46): 20–29: 47.8% 30–39: 21.7% 40–49: 28.3% 50+: 2.2%	100% male	80.4% White, 15.2% Black, 4.3% Asian	NR	Of the 12 who were screened using the ADOS, only 2 were known to the prison to have ASD (83.5% unidentified). 8/37 with positive ASD traits and 1/11 ADOS confirmed met criteria for alcohol dependency. There were no sig. differences between groups. There was no reported alcohol abuse across both ASD traits and ADOS confirmed groups. ASD positive trait sample had sig. more suicide-related behaviours and self-harm compared to prisoners with no neurodevelopmental difficulties. However, there were no differences in the ADOS-confirmed sample. In terms of comorbid mental health problems compared to no neurodevelopmental difficulties: ASD positive traits were sig. different across depression, major depression with psychotic features, mania or hypomania, generalised anxiety disorder, social phobia, obsessive compulsive disorder (OCD) and antisocial personality disorder. In the ADOS confirmed group, social phobia and OCD were sig. different from controls.
Haw et al (2013)^72^	40%	Specialist, low- secure psychiatric unit for adults	Matched cohort, quantitative non-randomised (*n* = 88; ASD *n* = 45, control *n* = 43)	Retrieved from electronic case notes (ICD-10 criteria)	ASD: Mdn = 27 (NR) 19–57	100% male	ASD: Ethnicity: 88.9% white British, 11.1% other ethnic group	ASD: Educational support: 53.5% yes, 46.7% no; History of childhood abuse/neglect: 22.2% yes, 77.8% no; No school qualifications: 66.7% yes, 33.3% no; previous employment: 44.4% yes, 55.6% no; marital status: 97.8% single, 2.2% married/divorced; children: 6.7% yes, 93.3% no	Prevalence of alcohol use or dependence was sig. lower in ASD compared to controls. Lifetime history of alcohol abuse was lower, but not sig., in ASD compared with controls. Intoxicated at the time of index offence was lower in ASD but not sig. different than controls.
**Population-based cohorts**									
Abdallah et al (2011)^73^	60%	Danish nationwide health registers	Matched cohort, quantitative non-randomised (*n* = 1234; ASD *n* = 414, control *n* = 820)	Register dx based on ICD-8, ICD-10 codes	ASD: M = 16.28 (4.55) NR	ASD: 80.9% male	NR	NR	Lower prevalence of alcohol-related disorders (ARD) in ASD compared to controls. Non sig. crude odds ratio and adjusted odds ratio of ASD having a lower risk of having ARD compared to controls.
Butwicka et al (2017)^74^	80%	Swedish longitudinal, population-based registers	Longitudinal matched cohort, Quantitative non-randomised (*N* = 1 376 286; ASD *n* = 26 986; controls *n* = 1 349 300)	Registers dx based on ICD-8, ICD-9 and ICD-10 codes	ASD: Year of birth: 17.4% 1970–79; 49.5% 1980–89; 33% 1990–99; (18–47)	ASD:70.4% male	ASD: Mother's region of birth: 82.8% Sweden, 4.2% other Nordic, 12.8% outside Nordic, 0.1% unknown. Father's region of birth: 81.8% Sweden, 3.8% other Nordic, 13.8% outside Nordic, 1.2% unknown	ASD: Family income (percentile): 23.7% < 20; 71.5% 20–79; 4.8% ≥ 80. Education: 59.3% Primary and lower; 31.6% upper secondary; 5.9% post-secondary; 3.2% postgraduate	Probands had a substantially increased risk of somatic disease linked to alcohol use. AUD was the third highest-risk substance. Sig. difference between ASD and non-ASD controls for Crude OR which was maintained when adjusted for parental education, family income and SUD dx before ASD dx. Sig. difference within ASD of AUD dx compared to non-ASD when using different International Classification of Diseases systems (ICD-8/9 *v.* 10). Risk of AUD in ASD compared to non-ASD controls in descending risk order of comorbidity: ADHD, ADHD + intellectual disability (ID), none, ID. Risk order is maintained when accounting for additional multivariate analysis. Risk of AUD in ASD related to non-ASD controls, who received a neuropsychiatric disorder dx prior to SUD, descending risk order of comorbidity: none, ADHD, ADHD + ID, ID. Risk order is maintained when accounting for additional multivariate analysis. Shared AUD liability between ASD group and relatives, descending highest odds ratio: parents, half-siblings, full siblings.
Chen et al (2017)^75^	60%	Taiwan's National Health Insurance (NHI)	Matched cohort, quantitative non-randomised cohort (*N* = 28 090; ASD *n* = 5618, control *n* = 22 472)	Medical records using dx codes from ICD-9-CM.	ASD: Age at enrolment M = 17.2 (4.58) 12–29	ASD: 78.2% male	NR	ASD: Taiwan urbanisation: 18.7% 1 (most urbanised), 30.4% 2, 9.5% 3, 8.6% 4, 32.8% 5 (most rural). Income-related insurance amount (NTD, New Taiwan Dollars): 86.5% ≤ 15 840 /mo, 12.4% 15 841–25 000 /mo, 1.1% ≥ 25,001 /mo	Prevalence of AUD in the ASD sample was non-sig. different from controls. Cox regression analysis found autistic males with AUD were more likely to attempt suicide, stratified by age and gender, during follow-up compared to controls. Overall, AUD in ASD was related to a sig. increase of suicide attempts compared to controls.
Croen et al (2015)^76^	80%	Kaiser Permanente Northern California integrated healthcare insurance	Matched cohort, quantitative non-randomised cohort (*N* = 16 577, ASD *n* = 1507; control *n* = 15 070)	Medical records using dx codes from ICD-9	ASD: M = 29.0 (12.2) 18–65+	ASD: 73.1% male	ASD: 65.6% White, 3.9% Hispanic, 7.6% Black, 11.1% Asian, 11.7% Other	ASD: Type of insurance: 73.5% Kaiser Permante, 24.9% Medicaid, 1.7% self-pay	Sig. difference between ASD adults and controls for the prevalence of harmful alcohol use and dependence. Self-reported alcohol use was lower in the ASD group. Autistic females were diagnosed less often than men for alcohol abuse and dependence. Higher odds ratio for harmful alcohol use and alcohol dependence across male and female ASD participants compared to controls.
Hermens et al (2013)^77^	60%	Youth Mental Health cohort	Cross-sectional, quantitative non-randomised cohort (*N* = 2112; ASD *n* = 22; Alcohol Use Disorders Identification Test (AUDIT) sample *n* = 522, of AUDIT sample, ASD *n* = 5	Clin. ax. using DSM-IV criteria, WHO-ASSIST, AUDIT	ASD (*n* = 5) M = 15.8 (2.3) 12–30	ASD: 100% male	NR	NR	The proportion of weekly alcohol use in the total sample: 25% (*n* = 1/4) women and 5.5% (*n* = 1/18) men. Prevalence of AUDIT categories within the total ASD sample: Abstainers 13.64%, low risk 4.5%, high risk 4.5%. Prevalence of AUDIT categories within samples with ASD: Abstainers 1.89%, low risk 0.56%, high risk 2.17%.
Huang et al (2021)^78^	60%	Taiwan National Health Insurance Programme	Retrospective, matched cohort, Quantitative non-randomised (*N* = 32 995, ASD *n*= 6599, controls *n* = 26 396)	Medical records using dx codes from ICD-9-CM criteria	ASD: M = 11.9 (5.1) (<6–>18)	ASD: 77.2% male	NR	ASD: Years of education: 15.5% ≥ 12; Marital status – married: 1.7%; Level of care: 45.9% Hospital centre, 47.5% Regional hospital, 6.7% Local hospital; Charlson Comorbidity Index (CCI): 93.5% 0, 6.1% 1, 0.4% ≥ 2; Urbanicity of residence: 45.8% 1 (highest), 40.1% 2, 13.4% 3, 0.7% 4; Monthly income: 73.7% < 18,000, 16.3% 18 000–34,999, 10% ≥ 35 000	Adjusted hazard ratios (aHRs) for AUD were sig. higher for the ASD group than non-ASD controls. Crude incidence of AUD per 100,000 person-years was higher in the ASD group in comparison to non-ASD controls. Subgroup analysis of AUD ASD subgroups aHRs receiving one psychotropic agent and multiple psychotropic agents were lower than the group receiving no psychotropic agents. 8 psychiatric comorbidities were found to be associated with an increase in aHRs for AUD compared to the absence of these comorbidities within the ASD group compared to non-ASD controls. Comparing these comorbidities with the non-ASD sample, aHRs were substantially higher in ASD patients with either anxiety disorder or impulsive control disorder.
Langley et al (2023)^79^	60%	Secure Anonymised Information Linkage (SAIL) databank	Matched cohort; Quantitative non-randomised (*N* = 43 698, ASD *n* = 5001, ASD controls *n* = 11 427, ADHD *n* = 7738, ADHD controls *n* = 19 532)	Medical records using ICD-10 and NHS READ codes	ASD (end of follow-up): M = 19.4 (2.6) NR	ASD: 78.9% male	NR	ASD: Welsh Index of Multiple Deprivation (WIMD) quintile (start of follow-up): M = 3.2 (1.4) 1 = 15% 2 = 16% 3 = 19% 4 = 24% 5 (most deprived areas) = 32%	In the total sample, 0.9% (*n* = 5001) had ASD, with diagnosis more likely in men (1.5%) than women (0.4%). Similar levels of AUD to matched controls. These associations were robust when controlling for gender, record availability and deprivation. Higher Cox regression model for males compared with women.
Roux et al (2022)^80^	80%	Medicare & Medicaid Services across a range of clinical settings	Matched cohort, quantitative non-randomised cohort (*N* = 2 892 718; ASD only *n* = 209 795; ID only *n* = 790 719; ASD + ID *n* = 123 705; Control *n* = 1 768 499)	Medicaid data using dx codes based on ICD-9 criteria	ASD + SUD only: 51.4% 11–17, 24.19% 18–29, 11.02% 30–45, 13.39% 46–64. ASD, ID, + SUD: 35.82% 11–17, 32.29% 18–29, 16.13% 30–45, 15.76% 46–64.	ASD + SUD 71.46% male ASD, ID + SUD 71.55% male	ASD + SUD:64.8% White, 18.7% Black, 1% Asian/Pacific Islander, 7.2% Hispanic/Latino, 8.4% Other. ASD, ID, + SUD: 62% White, 21% Black, 1.6% Asian/Pacific Islander, 7.3% Hispanic/Latino, 8% Other	Medicaid enrolment: ASD + SUD: 32.77% poverty, 55.5% disability, 11.73% other ASD, ID, + SUD: 8.07% poverty, 85.84% disability, 6.1% other	SUD – Alcohol type: all groups were sig. different across chi-square tests for ASD only, ASD and ID, and controls. Overall AUD prevalence across ASD samples 1.45%.
Underwood et al (2019)^81^	40%	Welsh National Centre for Mental Health	Matched cohort, quantitative non-randomised cohort (*N* = 181; ASD *n* = 105, control *n* = 76)	Case-note review using ICD-10 criteria	ASD: M = 37.8 (12.3)	ASD: 76.2%	100% Caucasian	ASD: Employment: 32% currently working, 49.5% not currently working due to sickness or disablement; Marital status: 50.5% married or cohabiting	9.5% (*n* = 10) met ICD-10 criteria for alcohol use disorder and 36.2% had problems due to alcohol use (*n* = 21). Sig. increase alcohol-related problems in ASD sample compared to controls.
Yu et al (2019)^82^	80%	Swedish longitudinal, population-based registers	Matched cohort, quantitative non-randomised cohort (Major sample with controls *n* = 3 859 497; ASD *n* = 9,529, ASD controls *n* = 186 017)	Dx from inpatient or outpatient setting based on ICD-10 criteria	ASD: Follow-up start age: M = 17.7 (10.4)	100% male	NR	39.5% low income, 99.3% single, 8.4% born abroad, previous intimate partner violence (IPV) 0.2%	The crude hazard ratio of perpetrated IPV was higher in the ASD sample with comorbid AUD compared to non-AUD. Sig. adjusted hazard ratio perpetrated IPV against women by men with ASD and comorbid AUD. Non sig. hazard ratio of perpetrated IPV against women by men with ASD without comorbid AUD. Sig. ratio of hazard ratios of perpetrated IPV against women in men with mental health diagnoses, between adjusted hazard ratios, after adjustment of prior alcohol use disorder, between individuals with mental health disorders and unaffected siblings.

ADHD, attention-deficit hyperactivity disorder; aHRs, adjusted hazard ratios; ARD, alcohol-related disorders; ASD, autism spectrum disorder; AUD, alcohol-use disorder; AUTS2, autism susceptibility candidate 2 gene; CBT, cognitive behavioural therapy; Clin ax., clinical assessment; dx, diagnosis; GAD, generalised anxiety disorder; ICD, International Classification of Diseases; ID, intellectual disability; M, mean; Mdn, median; NHI, national health insurance; NR, not reported; NTD, New Taiwan Dollar; OCD, obsessive compulsive disorder; SUD, substance-use disorder; WIMD, Welsh index of multiple deprivation.

Measures: ADOS, Autism Diagnostic Observation Schedule,^83^ adapted for prison use (89); AQ, autism-spectrum quotient (90); AUDIT, Alcohol Use Disorders Identification Test (87); CCI, Charlson Comorbidity Index (91); Hollingshead scale, Hollingshead four factor index of social status (92); Taiwan urbanisation (93).

a. Quality assessment summarised by Mixed-Methods Appraisal Tool^58,59^ total domain score. Percentage of quality criteria met are presented as star (*) ratings: *****, 100%, ****, 80%, ***, 60%, **, 40%, *, 20%.

b. Age is reported in years. Mean, (s.d.) and (range) reported where available.

c. Socioeconomic variables reported for ASD samples only where available. In absence, whole sample characteristics were reported. Variables include: education, employment, income, living arrangement, marital status, social support.

## Results

**Fig. 1:**
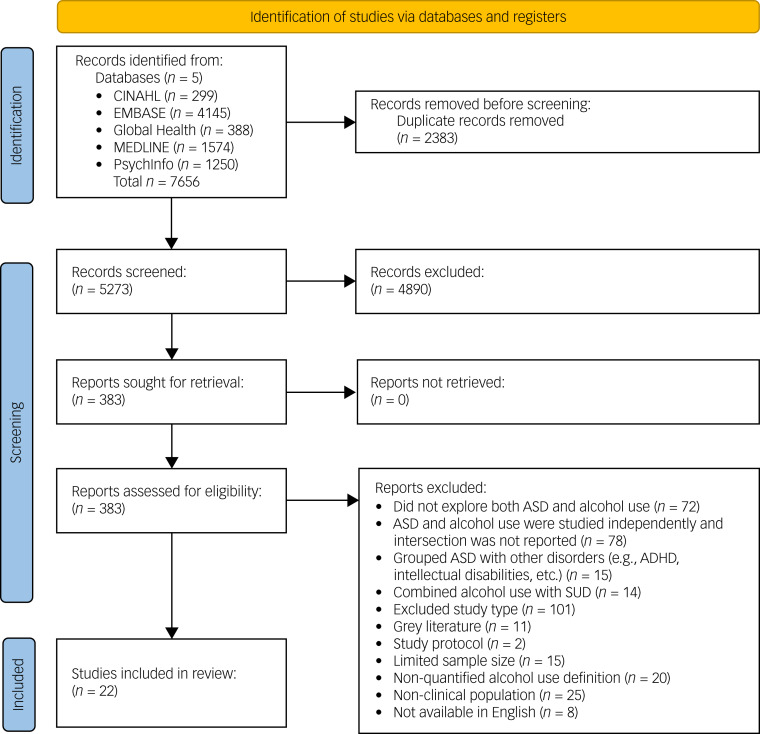
PRISMA flow diagram. ADHD, attention-deficit hyperactivity disorder; ASD, autism spectrum disorder; SUD, substance-use disorder.

Twenty-two studies were included in the final review following the full-text screening of 383 articles (Fig. 1). Full study characteristics and a summary of individual findings are presented in Table 1. Detailed findings are reported below, and the data are synthesised into main components for aetiological risk factors and implications for co-occurring AUD in ASD in Box 1 as a concept map.^58^ Four components of biological, psychological and social/environmental aetiological risk factors emerged alongside ten potential implications of co-occurring AUD in ASD.

### Study characteristics

The publication year of included studies ranged from 2003 to 2023, with 45% of studies being published in the past 5 years (2019 to 2023). Studies were primarily conducted in European countries (*n* *=* 13), followed by the USA (*n* *=* 4), Japan (*n* *=* 2), Taiwan (*n* *=* 2) and Australia (*n* *=* 1). Study designs consisted of 20 quantitative (case-control *n* *=* 12, cross-sectional *n* = 5 and cohort studies *n* = 3) and two qualitative (interviews *n* = 2*)*. Overall, samples were derived from a range of settings: drug and alcohol services (*n* *=* 5), ASD services (*n* *=* 3), a combination of both ASD/AUD services (*n* *=* 1), forensic (*n* *=* 3) and large, population-based registers (*n* *=* 10).

### Quality appraisal

Overall, the average quality score of included studies was 66%, with the majority of studies 60% or above (*n* = 17). Studies that scored below 60% were retained due to limited available literature. The main quality issues within clinical settings were insufficient descriptions of study samples, utilisation of non-validated measures and absence of controls for confounding variables. In large population-based studies, diagnoses were primarily sourced from medical records, with a large proportion of missing data, and potential recruitment bias within samples due, for example, to paid healthcare insurance.

#### Participant characteristics

A total of 750 ASD participants were represented from clinical settings and 389 281 from large population registers. The pooled mean of ASD gender was 83.4% male. The ages of included participants ranged from 1.1 years to 85 years, with a median of mean 32.2 years (s.d. = 14.58) reported by 16 (72.7%) studies. Of studies that reported socioeconomic status (count 16) and race and ethnicity (count 8), records were incompatible for comparison across categories. For studies that reported demographics, participants were primarily White/Caucasian, achieved primary and secondary education qualifications, were unemployed, single, possessed lower socioeconomic scores and lived in urbanised areas (see Table 1 for individual breakdown).

#### Prevalence and risk

Excluding studies with exclusive ASD-AUD samples (i.e. samples that only included individuals with both by deliberate design), prevalence in clinical settings ranged from 6.7% to 39%; while in large population-based registers, the rate varied between 0.7% and 9.5%. Pooled prevalence was higher for clinical settings (121/750, 16.13%) than large population registers (6124/389 281, 1.57%).

In large population-based registers, the associated average risk of AUD within ASD patients compared with non-ASD controls, varied significantly. Two studies suggested a decreased AUD risk,^76,83^ three studies found similar or no difference in AUD risk,^73,75,79^ and four studies indicated increased AUD risk.^74,78,80^

#### Aetiological risk factors

Risk factors involved in the development of AUD in ASD included genetics, co-occuring conditions, gender and age.

Three studies of varying quality found evidence for a genetic pattern and increasing average risk between ASD and AUD.^63,66,74^ As both conditions are heterogeneous in origin, a lack of genetic specificity within the literature can only conclude an overall association. The indicated risk of AUD is substantially compounded by co-occurring disorders, such as attention-deficit hyperactivity disorder (ADHD).^68,74,78^ However, in comparison with ADHD alone, ASD was associated with a lower average risk of AUD.^68^ Furthermore, when compared with non-ASD controls, ASD-AUD patients present with higher occurrences of anxiety disorders accentuating the complexity between disorders.^71,78^ Gender differences contribute to this complexity, as autistic women were underrepresented and diagnosed less frequently than autistic men in included studies.^66,76,79^ Yet findings present a higher risk for AUD in autistic women, indicating a nuanced relationship given the unequal distribution across samples and overlapping confidence intervals with male counterparts, which requires further study. In addition, there was an emerging pattern of alcohol use developing in older ASD patients when compared with controls.^67,68^

Two qualitative studies explored the development and everyday life consequences of SUD in ASD.^62,69^ Emerging themes indicated two interrelated alcohol use coping motives: self-medication and social facilitation. Proactive strategies position alcohol use as a means to self-medicate symptoms associated with cognitive and emotional distress, as well as idiosyncratic features of ASD such as sensitivity to sensory processes.^62,69^ Reactive strategies occur in the context of social situations, to facilitate interactions or cope with associated negative appraisals. ASD patients described the inability to express themselves and anticipated social rejection, leading to feelings of loneliness and social exclusion.^62,69^ Through alcohol use, insecurities and oversensitivity can be mitigated by feeling more confident and comfortable in social situations.^62,69^ This reinforces the use of alcohol due to elevated mood, social gains, connection and alleviation of social anxiety.^62,69^

#### Implications of alcohol use

From this review, the literature suggests a continued pattern of alcohol use could impact general functioning and increased harmful experiences, such as somatic disease, intimate partner violence and suicide attempts.^62,74,75,82^ Further analysis of suicide attempts, stratified by age and gender, found only autistic men had a significant increase in average risk, compared with controls, lending further support to gender differences.^75^ The included study of intimate partner violence only investigated the perpetration against women by men with comorbid ASD and AUD. Hazard ratios of engaging in intimate partner violence against women by men were significantly greater in autistic people with comorbid AUD.^82^

The consequences of alcohol-related problems can be maintained by specific ASD traits.^61^ A series of exploratory Pearson chi-square tests found traits of rigidity, social avoidance and withdrawal, were significantly related to harmful drinking patterns. For example, rigidity in thought could create a barrier to implementing lifestyle change. The authors questioned whether these specific traits were indicative of long-term difficulties, such as anxiety and inflexibility to adapt to social cues or the overlap of these traits in the spectrum of alcohol use; although ASD traits were not found to be related to rates of abstinence at follow-up.^61,65^ If certain characteristics of ASD sustain harmful patterns of alcohol use, current intervention methods may not adequately address patient needs, and greater accommodation for ASD traits may yield greater outcomes.^61^ As such, adapted ASD-AUD treatment, with a focus on core ASD symptoms, social skills and ASD-related stress, presented promising results requiring further study.^64^

Box 1 Concept map of findings for alcohol use in clinical, autistic populations. ASD, autism spectrum disorder; AUD, alcohol-use disorder; ADHD, attention-deficit hyperactivity disorder.

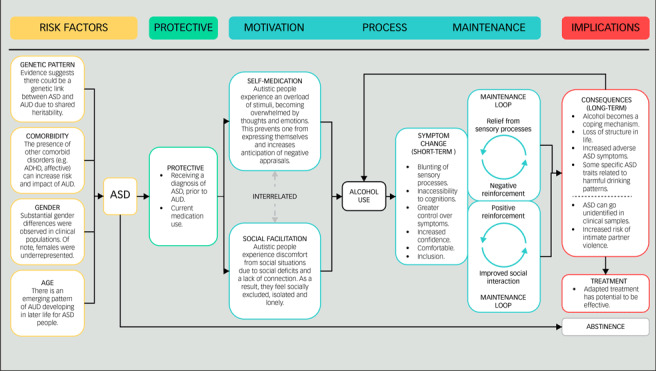

Potential protective factors of developing AUD in ASD included psychotropic medication use and a prior diagnosis of ASD. Huang et al^78^ found a reduction in overall AUD average risk if autistic patients had received one or multiple psychotropic medications, even in the context of additional co-occurring disorders. Psychotropic medication was classified as antidepressants, second-generation antipsychotics and mood stabilisers. The risk of AUD was decreased among patients who had received a prior diagnosis of ASD, for both patients with no co-occurring conditions and co-occurring ADHD.^74^ However, one forensic study found the majority of cases that met ASD diagnostic criteria, were previously unknown to providers.^71^

## Discussion

This narrative systematic review aimed to identify relevant clinical studies, explore the aetiology and implications of alcohol use in ASD and suggest future research, policy and clinical recommendations. The final review included 22 studies, extracting data from healthcare settings and large, population-based registers. The findings of this review stress the variable nature of studies investigating AUD in ASD. Due to divergent quality and heterogeneous parameters, our overall findings are speculative.

Of the included studies, the prevalence of AUD in ASD appears to range between 1.6% in large population registers and 16.1% in clinical settings. Within global populations, irrespective of neurodiversity status, the lifetime prevalence of AUD is estimated to be 8.6%.^84^ This is substantially greater than the rate found in this review, potentially supporting the evidence that autistic populations generally report lower rates of alcohol use.^16–18^ This is further compounded given the low global prevalence rates of ASD,^7^ despite the recent suggested increase in ASD diagnoses.^4^ With regard to clinical settings, the observed prevalence in this review is greater than that of one study (11.8%), which compared rates of AUD in European primary care to a general population.^85^ However, Manthey et al^85^ did not differentiate for neurodiversity status. It is possible AUD is exacerbated by ASD symptoms, and given the higher observed rate in this review, screening within clinical services is important to explore the intersection between disorders and improve the precision of prevalence rates.

This review identified four possible risk factors for the development of AUD in ASD: age, co-occurring conditions, gender and genetics. Based on the broader literature and this review, both conditions may share genetic vulnerability.^32,63,66,74^ Although specific genes are not clearly identified, studies considered family history and ancestry.^66,74^ Adopting an epigenetic viewpoint, considering parental monitoring and alcohol use can influence the development of AUD,^36,39,46^ it would be interesting to investigate the associations between genetics, family environment and AUD development across the severity and lifespan of ASD.

Findings suggest the risk of AUD increases with age in ASD and in the presence of co-occurring difficulties in clinical samples, an observation found in other literature within non-clinical samples.^18^ However, the risk of AUD could be decreased with a prior diagnosis of ASD.^74^ We hypothesise that a prior diagnosis of ASD explains some daily life experience and potential availability for support to manage symptoms. In the absence of appropriate support, ASD patients may seek other means to manage or alleviate symptoms. This may also offer an explanation as to why the risk of AUD was reduced for ASD populations receiving psychotropic medication.^78^

Alcohol use may lead to reduced sensory perception and inhibit cognitive processing, such as accessibility to self-critical memories, leading to greater symptom control.^62,69^ Such motivations have been explored in non-clinical ASD samples yielding similar results.^16,48–51^ In comparison with general populations, motivations for alcohol use can fall into two categories: enhancement and coping.^86^ Both of these predictors can lead to alcohol use problems. Yet, using alcohol to cope can lead directly to alcohol use problems, whereas enhancement is indirectly associated with use through alcohol use problems. This is comparable with the interrelated factors of self-medication (coping) and social facilitation (enhancement) found in this review for ASD-AUD.^62,69^

The processes of how harmful alcohol use develops in ASD are unclear. Within wider literature, Cho et al^87^ found longitudinal associations for two reinforcement cycles related to alcohol dependence, with a stronger association for negative reinforcement. In the context of ASD and this review, improved social interaction (positive) and relief from sensory processes (negative) could form reinforcing maintenance loops (see Box 1). Therefore, the function of alcohol use for ASD people could influence how AUD develops and provide a theoretical target for intervention. However, existing approaches would require appropriate adaptation to reduce barriers, create shared understanding and meet specific ASD population needs.^47^

### Principles of care and research

The findings of this review highlight the significant need for research to improve clinical practice for ASD-AUD patients. In Table 2, we have provided guiding principles for clinicians and researchers to consider and take forward, based on the review findings. Seven principles are outlined across assessment, consideration for co-occurring difficulties and life course, prevention, function of use, education and training and adapted treatment. We offer a further four novel research recommendations.

**Table 2:** Seven guiding principles related to aetiology and implications of alcohol use and autism. Included recommendations for clinical practice and novel research ideas.

Domain	Guiding principle	Recommendation
Assessment	AUD in ASD is not routinely assessed within clinical services or research using universal, standardised measures. Autistic people usually receive a diagnosis later in life, increasing potential harm from alcohol use.	Clinical: Screen for alcohol use in ASD using gold standard measures (e.g., ASSIST-Lite). Measure alcohol use using standardised measures (e.g., AUDIT). Use of existing ‘gold-standard’ assessments for ASD (e.g., ADOS) and AUD (e.g., AUDIT or SCID) for diagnosis. Research: Establish minimum dataset and measures to use in ASD and AUD research. Development of a diagnostic tool to assess AUD in ASD, accounting for the severity of ASD, harm from AUD and motivations for alcohol use. This tool would benefit from an alternative approach to existing measures based on gender-specific criteria.
Co-occurring difficulties	Patients who have psychological comorbidities, such as ADHD, ID or low mood, increases the risk of AUD in ASD.	Clinical: Patients should be offered a comprehensive assessment accounting for co-occurring mental health difficulties. Working alongside co-occuring conditions. Targeted treatment for the function of alcohol use or most influential comorbid disorder (e. ADHD). Research: Impact of comorbidity on development and life course of AUD in ASD.
Life course	Emerging pattern of AUD developing later in life for ASD patients.	Clinical: Clinicians should screen for ASD and hold ‘sensible’ conversations with AUD patients. Appreciation of neurodiversity as a consideration of the patient's story. Existing services should develop links across ASD and drug and alcohol services to increase joined-up care. Research: Risk factors, triggers and facilitators in the development of AUD. Timing of diagnosis and impact on life (potential harms).
Prevention	Normalising the attitudes and beliefs of alcohol use in younger ASD patients.	Clinical: To educate neurodiverse young adults on the associated risks of alcohol use. To provide realistic expectations as an aspect of education from a non-stigmatising stance. Early intervention for emerging adulthood ASD and AUD patients. Adapted to allow for time for generalisation-flexibility in the length of time to see a client. Research: Development of co-produced programmes designed to educate neurodiverse young adults on alcohol use and reduce stigma based on behavioural principles.
Function of use	The use of alcohol for social facilitation or self-medication is more likely to reinforce harmful use.	Clinical: Bespoke formulation of motivators and functions-including specific ASD experiences. Develop healthy coping strategies. Research: Research investigating the link between social facilitation and self-medication. Consideration for severity and cross-spectrum differences within ASD and AUD.
Adapted treatment	Adapted treatment has the potential to be effective. Features to include an aim to increase a sense of control in daily life, and change dysfunctional beliefs, and coping strategies.	Clinical: Emphasis on understanding how autism impacts the patient (function/features) and adapting interventions. These considerations extend to alcohol-based interventions such as detox or rehabilitation. Creating neurodiverse friendly environments (quiet zones etc). Proactive role in intervention planning such as providing extra time for therapy or support to access ‘safe’ recovery spaces e.g., AA for neurodivergence. Psychoeducation on possible connections between AUD and ASD. Coping with ASD-related stress (e.g., sensory overload). Research: Manualised intervention package to be tested. Efficacy in comparison to treatment as usual. Active ingredients. Use of buddies in treatment programmes.
Education and training	Neurodiverse patients have the potential to go unidentified in forensic populations.	Clinical: Staff training to understand how to identify and work with ASD. Increased support for forensic patients with ASD. A potential pathway for screening and referring to local, psychologically informed environments. Research: Development of a cross-clinical setting training package for working with ASD, with consideration for AUD.
Novel research recommendations: 1. Autistic traits did not impact abstinence rates or whether ASD and AUD patients changed consumption. • Exploratory research to understand why abstinence rates are not related to ASD. Initial findings suggest this is related to rigidity and social deficits. Factors which are associated with maintenance of harmful use. 2. Biomarkers for risk of AUD in ASD. • Following genetic patterns and early evidence for shared links between ASD and AUD. Additional biological and genetic research should be completed. 3. Family support. • To explore systemic approaches for change/skill development and to help implementation in social environments. Working alongside family, support networks and clinical teams. 4. Gender differences. • Females are underrepresented in existing research. There is a dearth of research on gender differences which requires examination.		

AA, alcoholics anonymous; ADHD, attention-deficit hyperactivity disorder; ADOS, Autism Diagnostic Observation Schedule;^83^ ASD, autism spectrum disorder; ASSIST-Lite, Alcohol, Smoking and Substance Involvement Screening Test short-form (86); AUD, alcohol-use disorders; AUDIT, Alcohol Use Disorders Identification Test (87); ID, Intellectual disability; SCID, Structured Clinical Interview for the DSM (88).

To outline some of our principles and recommendations, we draw on the findings of this review and the wider literature. As highlighted, autistic people may be accessing services undiagnosed,^71^ and if a timely diagnosis could protect autistic people from developing AUD,^68,74^ routine assessment could prevent future harm. However, as the waiting time for ASD assessment grows,^8^ current services could implement screening tools to inform clinical formulations. Hence, the development of a specific ASD-AUD screening tool may benefit future research. Subsequent studies should consider the overrepresentation of male participants, as indicated by this review, and the potential bias of some existing measures towards men.^83^ In addition, greater awareness is a promising sign of advocacy for the needs of autistic people, yet this does not necessarily translate to available services.^7^ Cross-service collaborations may prove fruitful for developing individual pathways to share resources. Similar to harm-reduction strategies, co-produced research, education and training could inform future prevention strategies.

A further interest is the emerging pattern that AUD develops later in life for autistic people.^67,68^ This poses the question of whether this could be attributed to the change in diagnostic criteria over time^74^ or to the limited resources to diagnose autistic adults.^8^ We could also question whether it could be a result of the function or motivation to use alcohol as a coping strategy, as availability increases in adulthood.^62,69^ Future longitudinal studies could explore the development of AUD and functions of use over time in autistic adults. This in turn could direct adapted treatment, such as one included study,^64^ and future randomised controlled trials to test for efficacy.

### Limitations

Due to the inconsistency of reporting demographics and severity of both spectrum disorders, comparisons between studies are difficult to establish. Without knowing the specificities across the spectrum of ASD with AUD present, it is likely to be difficult to meet the needs of this patient group adequately. The findings of this review should be taken with caution due to varied sample sizes, absence of control groups and lack of consideration for confounding variables. Furthermore, diagnoses sourced from medical records do not specify how assessments were conducted or which diagnostic tools were used. These issues are deepened by the differences in conceptualisation across classification manuals. In addition, this review focused on clinical samples only, excluding research on non-clinical samples, which could disregard existing applicable findings. Furthermore, the use of the MMAT to appraise the quality of studies may overlook methodological concerns that include more variables with non-standardised measures. None of the included studies were randomised controlled trials, and despite identifying possible associations, this review lacks the exploration of casual relationships.

This review, the first of its kind, highlights emerging trends and areas for future development in research and clinical practice. Included studies have identified some possible factors that may be associated with the development of AUD in ASD, yet further research is required. Future research would benefit from carefully defined variables, such as those identified by reviews like this, with the aim longitudinally to identify both causative factors and effective management strategies.

## Supporting information

Barber et al. supplementary material Barber et al. supplementary material

## Data Availability

Data availability is not applicable to this article as no new data were created or analysed in this study.
