# Supplementary material for: Alcohol use among populations with autism spectrum disorder: narrative systematic review
Source: BJPsych Open. 2025 Jan 13;11(1):e15. doi: 10.1192/bjo.2024.824 (PMC11736162; doi:10.1192/bjo.2024.824)
Supplement: Barber et al. supplementary material [file S205647242400824Xsup001.docx]

**Supplementary Materials**

| **Table 2: Mixed Methods Appraisal Tool (MMAT) ratings for each study with quality appraisal comments on strengths, weaknesses, and relevance to review** | | | | | | | | | | | |
| --- | --- | --- | --- | --- | --- | --- | --- | --- | --- | --- | --- |
| **Studies** | |  | **MMAT Criteria** | | | | | | **Comments** | | |
| Author | Year | Type | 1 | 2 | 3 | 4 | 5 | % | Main strengths | Main weaknesses | Study relevance to question |
| Hildebrand et al. | 2021 | 3. Quantitative non-randomised | 1 | 0 | 0 | 0 | 1 | 40 | • Long period of data collection (2004-2012) across two treatment centres with a clear description of the study sample, including exclusion criteria. • Differences between participating and non-participating groups compared. | • Autistic personality traits (APTS) were conceptualised as items on the Structured Clinical Interview of Personality Disorders (SCID-II) and Temperament and Character Inventory (TCI) to represent domains of Wing's Triad (unvalidated). • Significant drop-out at post-treatment (40.5%). • No method to control for confounders. | Study aimed to explore whether APTs were related to alcohol-use disorder (AUD) characteristics and their impact on treatment outcomes. |
| Kronenberg et al. | 2014 | 1. Qualitative | 1 | 1 | 1 | 1 | 1 | 100 | • Appropriate approach for study.  • Data reached saturation and findings consistent with quotes used.  • Wide range of topics to explore everyday behaviour across different life domains. |  | Studied patient perspectives of everyday consequences living with substance-use disorder (SUD) and co-occurring autism spectrum disorder (ASD). |
| Narita et al. | 2016 | 3. Quantitative non-randomised | 0 | 1 | 1 | 1 | 1 | 80 | • Clearly defined method for polymerase chain reaction for elucidating frequencies of polymorphism.  • Complete dataset.  • Case-control design with healthy controls. | • Poor description of sample with some data unavailable. | Autism Susceptibility Candidate 2 (AUTS2) gene is implicated in the development of ASD. Exploring genetic factors of AUD could illicit a link. |
| Walhout et al. | 2022 | 3. Quantitative non-randomised | 1 | 1 | 0 | 0 | 1 | 60 | • Clearly defined sample with similarities to other research ASD samples.  • Validated measures used to capture variables of interest. | • Significant discontinuation of participation at time 1 (47.4%) and time 2 (52.6%).  • No control group. | Adapted cognitive behavioural therapy (CBT) treatment for ASD and co-occurring SUD from an Addictions Treatment Centre. |
| Yoshimura et al. | 2022 | 3. Quantitative non-randomised | 1 | 1 | 1 | 1 | 1 | 100 | • Representative clinical sample.  • Validated measure.  • 19.6% drop-out.  • Use of multiple cox proportional hazard analyses to examine confounding variables. |  | Study sought to investigate comorbidities and neurodevelopmental characteristics of dependent AUD after hospital treatment. |
| Miles et al. | 2003 | 3. Quantitative non-randomised | 0 | 0 | 0 | 0 | 1 | 20 | • Participants with full data only. | • Family history interview to ascertain alcoholism.  • Of 333 referrals, only 167 had complete family history data.  • Some control comparisons but overall results do not account for confounders. | Use of family history method to determine the prevalence of alcoholism in ASD. |
| M. Roy et al. | 2015 | 3. Quantitative non-randomised | 0 | 1 | 1 | 0 | 1 | 60 | • Use of validated measures to explore study variables.  • Complete dataset.  • No changes in observations. | • Small sample that could represent milder forms of Asperger’s Syndrome due to the method of diagnosis.  • Confounders not accounted for. | Study examined comorbidities and the course of Asperger’s Syndrome across different areas of participant’s lives. |
| Yule et al. | 2023 | 3. Quantitative non-randomised | 1 | 1 | 1 | 1 | 1 | 100 | • Wide sample with limited exclusion criteria.  • Use of structured, systematic assessments to examine diagnoses.  • Minimal data loss with no changes in exposure.  • Two control groups accounting for confounders with related samples. | • Of note, study did not use validated clinical diagnostic measures such as ADOS/ADI-R. However, process of diagnosis from structured interview is detailed, accounting for rater reliability. | Investigates risk factors related to developing AUD within ASD, ADHD, and controls. |
| Clarke et al. | 2016 | 1. Qualitative | 0 | 1 | 1 | 1 | 1 | 80 | • Consistent data collection methods in line with general practice.  • Use of negative case analysis to ensure representation of experiences.  • Interpretation and analysis coherence. | • The sample was limited and did not reach saturation. Predominantly male and lacked diversity. | Explored whether the experiences of having Asperger Syndrome contributed to development of SUD and facilitative mechanisms. |
| Anckarsater et al. | 2008 | 3. Quantitative non-randomised | 1 | 0 | 0 | 0 | 1 | 40 | • Multiple forensic settings. | • Medical chart review of alcohol use.  • Missing data (group 3).  • Confounders not accounted for. | Prevalence of ASD in forensic institutions alongside an overview of co-existing problems and other clinical features. |
| Chaplin et al. | 2021 | 3. Quantitative non-randomised | 1 | 1 | 0 | 1 | 0 | 60 | • Clear inclusion/exclusion criteria.  • Wide range of measures used to assess.  • Matched within with non-positive ASD traits. | • Missing data.  • Change in screening tool during observation.  • Literature on the use of screening tools with prisoners is limited. | Investigated ASD vulnerabilities and methods of screening in a prison population. |
| Haw et al. | 2013 | 3. Quantitative non-randomised. | 0 | 0 | 1 | 0 | 1 | 40 | • Limited missing data (*n = 6).*  • No changes in patient status. | • Tertiary referral service preventing generalisation.  • Majority of diagnoses retrieved from clinical notes.  • A control sample was used but was significantly different to the ASD group. | To describe characteristics of adult male ASD patients compared to non-ASD controls admitted to low-secure units. |
| Abdallah et al. | 2011 | 3. Quantitative non-randomised | 1 | 0 | 1 | 1 | 0 | 60 | • Nationwide dataset.  • Complete dataset.  • Frequency-matched cases based on gender and year of birth. | • Short study follow-up period.  • Coded diagnoses in databases using two different International Classification of Diseases systems (ICD-8/10). | Brief report to estimate psychiatric comorbidity rates of ASD |
| Butwicka et al. | 2017 | 3. Quantitative non-randomised | 1 | 0 | 1 | 1 | 1 | 80 | • Large, representative sample.  • Complete outcome data.  • Stratified regression models accounting for sex, birth year, and country of birth.  • Multivariate analyses adjusted for family income, parental education, and country of origin. | • Coded diagnoses in databases. | Investigates the risk of alcohol-related problems in ASD and associated comorbidities. |
| Chen et al. | 2017 | 3. Quantitative non-randomised | 0 | 0 | 1 | 1 | 1 | 60 | • Complete outcome data.  • Matched control sample (1:4 ratio) based on age, sex, and time of enrolment. | • Help seeking sample only.  • Diagnosis retrieved from medical records (diagnosed by board-certified psychiatrists). | Explores the risk of suicide of young adults with ASD considering confounding factors such as alcohol use. |
| Croen et al. | 2015 | 3. Quantitative non-randomised | 1 | 0 | 1 | 1 | 1 | 80 | • Large, ethnically diverse study population.  • Complete outcome data.  • Multivariate, logistic regression model controlled for sex, age, race/ethnicity. | • Non-validated diagnoses. | Determines prevalence of psychiatric and medical conditions among large population of ASD sample across ages. |
| Hermens et al. | 2013 | 3. Quantitative non-randomised | 1 | 0 | 0 | 1 | 1 | 60 | • Large sample across five years.  • Use of logistic regressions to account for diagnosis and age. | • Diagnosis made by variety of different assessing professions.  • Only sub-sample of participants completed self-report alcohol measure. | Determined rates of alcohol use in young people entering mental healthcare. |
| Huang et al. | 2021 | 3. Quantitative non-randomised | 0 | 0 | 1 | 1 | 1 | 60 | • Randomly selected control at 1:4 ratio, matched by sex, age, and index date.  • Adjusted hazard ratios accounting for a wide range of variables. | • Relies on medical records for diagnosis.  • Relatively small sample in comparison to original cohort, with a proportion excluded due to original diagnosis date and missing data. | Explores risk of SUD, associated comorbidities, and mortality risk amongst ASD patients compared to non-ASD controls. |
| Langley et al. | 2023 | 3. Quantitative non-randomised | 1 | 0 | 0 | 1 | 1 | 60 | • Large, representative sample.  • Matched control sample.  • Feasibility tested. | • Diagnosis drawn from medical records.  • One of the combined databases had 19% missing data. | To establish the feasibility of a nationwide e-cohort of ADHD and ASD for future longitudinal research. |
| Roux et al. | 2022 | 3. Quantitative non-randomised | 1 | 0 | 1 | 1 | 1 | 80 | • Very large sample of Medicaid enrollees.  • Sample matched to control sample by same-age enrolees. | • Diagnosis drawn from medical records. | To characterise the population of ASD, ASD+ID with and without SUD to estimate the prevalence of SUD and adjusted risk. |
| Underwood et al. | 2019 | 3. Quantitative non-randomised | 0 | 0 | 0 | 1 | 1 | 40 | • Use of a control group. | • Recruitment bias.  • Use of non-validated measures.  • Missing data. | Examines demographic, social, psychiatric, and physical health characteristics of the cohort presenting with ASD in adulthood compared with controls. |
| Yu et al. | 2019 | 3. Quantitative non-randomised | 1 | 0 | 1 | 1 | 1 | 80 | • Large, varied sample.  • Minimal missing data.  • Confounders accounted for in regression analysis and comparisons across demographics e.g. income, marital status, immigration status etc. | • Diagnosis drawn from medical records. | To investigate the risk of IPV against women among men, including ASD and SUD. |
